# Role of Autophagy in Lysophosphatidylcholine-Induced Apoptosis of Mouse Ovarian Granulosa Cells

**DOI:** 10.3390/ijms23031479

**Published:** 2022-01-27

**Authors:** Si Yang, Jie Chen, Bingchun Ma, Jinglei Wang, Jiaxiang Chen

**Affiliations:** 1 Department of Physiology, School of Basic Medical Sciences, Nanchang University, Nanchang 330006, China; yangsi1800@163.com (S.Y.); jchen1007@163.com (J.C.); mabc13@163.com (B.M.); 2 Jiangxi Provincial Key Laboratory of Reproductive Physiology and Pathology, Nanchang 330006, China

**Keywords:** lysophosphatidylcholine, oxidative stress, autophagy, apoptosis, ovarian granulosa cells

## Abstract

Lysophosphatidylcholine (LPC), also known as lysolecithin, is one of the major components of oxidized low-density lipoproteins (ox-LDL). In the pathogenetic process of diverse diseases, LPC acts as a significant lipid mediator. However, no evidence shows that LPC can affect the female reproductive system. In our study, we found that LPC inhibited the cell viability of primary mouse ovarian granulosa cells. Meanwhile, LPC was shown to induce apoptosis, which is accompanied by an increase in apoptosis-related protein levels, such as cleaved caspase-3, cleaved caspase-8 and Bax, as well as a decrease in Bcl-2. The total numbers of early and late apoptotic cells also increased in the LPC-treated cells. These results indicated that LPC could induce apoptosis of mouse ovarian granulosa cells. Furthermore, the increase in autophagy-related protein levels and the number of autophagic vesicles suggested that LPC could induce autophagy. The inhibition of oxidative stress by N-acetyl-L-cysteine (NAC) could rescue the induction of apoptosis and autophagy by LPC, which indicated that oxidative stress was involved in LPC-induced apoptosis and autophagy. Interestingly, the inhibition of autophagy by 3-MA could reserve the inhibition of cell viability and the induction of apoptosis by LPC. In conclusion, oxidative stress was involved in LPC-induced apoptosis, whileautophagy of mouse ovarian granulosa cells and the inhibition of autophagy could alleviate LPC-induced apoptosis.

## 1. Introduction

Lysophosphatidylcholine (LPC), also known as lysolecithin, is a major component of oxidized low-density lipoprotein (ox-LDL) and one of the phospholipids of eukaryotic cells. In view of the fact that LPC is converted from phosphatidylcholine (PC) under the effect of phospholipase A2 (PLA2), the content of LPC would be increased when the activity of PLA2 enhances [1]. Conversely, LPC can be converted into PC in the presence of acyl-CoA, which effectively scavenges excess LPC to maintain a relatively stable level in vivo [2]. A large number of studies have shown that abnormally elevated levels of LPC are harmful to normal cells [3]. Excessive binding of LPC tothe cell membrane will disrupt the membrane phospholipid bilayer and alter its structure and function, which leads to cell dysfunction and induces cytotoxic changes [4,5].

As a pro-inflammatory lysophospholipid, LPC participates in the regulation of immune cells and the immune response during inflammation, and induces the production of inflammatory factors, thereby causing damage to a variety of cells [6]. Studies have shown that the activation of calcium-independent phospholipase A2, mediated by caspase-3,induces the production of LPC in apoptotic cells [7], and, as a chemical inducer, LPC can induce apoptotic cells to release signals to initiate their own clearance by attracting phagocytes [8]. LPC has been reported to induce apoptosis and oxidative stress in vascular smooth muscle cells, endothelial cells, H9c2 cells and adipocytes [9,10,11,12]. However, no evidence shows that LPC can cause female reproductive toxicity.

The ovary is an important organ in the female reproductive system, and its most important function is the growth and development of follicles [13]. Ovarian granulosa cells wrap on the surface of the follicles and participate in the formation and development of follicles, which maintain ovarian function by secreting steroid hormones [14]. A number of studies have proved that follicular atresia results from the apoptosis of granulosa cells, which causes a decrease in the number of follicles [15,16]. As an important functional and structural unit of the ovary, ovarian granulosa cells are the target cells of various toxicants. Primary ovarian granulosa cell culture is a good system for evaluating ovarian function and oocyte quality, and has been widely used in the pharmacology and toxicology of reproductive system-related research.

Studies have shown that an increased LPC level can lead to demyelination of the nerve sheath and axonal degeneration, which is similar to the pathological changes in organophosphorus-induced delayed neurotoxicity (OPIDN), induced by tri-ortho-cresyl phosphate (TOCP) [17]. Our previous studies also found that TOCP can inhibit the activity of neuropathy target esterase (NTE) in mouse ovarian granulosa cells, and induce apoptosis and autophagy of ovarian granulosa cells [18]. As a hemolytic phospholipase, the physiological substrate of NTE is LPC [19], and hemolytic phospholipase is the main enzyme to remove LPC from the cell membrane. Therefore, when NTE activity is inhibited, the content of LPC is likely to increase [20], suggesting that the increase in LPC content might be related to the apoptosis and autophagy of mouse ovarian granulosa cells.

The toxic effect of LPC on the female reproductive system and its mechanisms has not been reported. This study intends to explore the toxic effect and mechanism of LPC on mouse ovarian granulosa cells.

## 2. Results

### 2.1. LPC Induces Apoptosis of Mouse Ovarian Granulosa Cells

**Figure 1:**
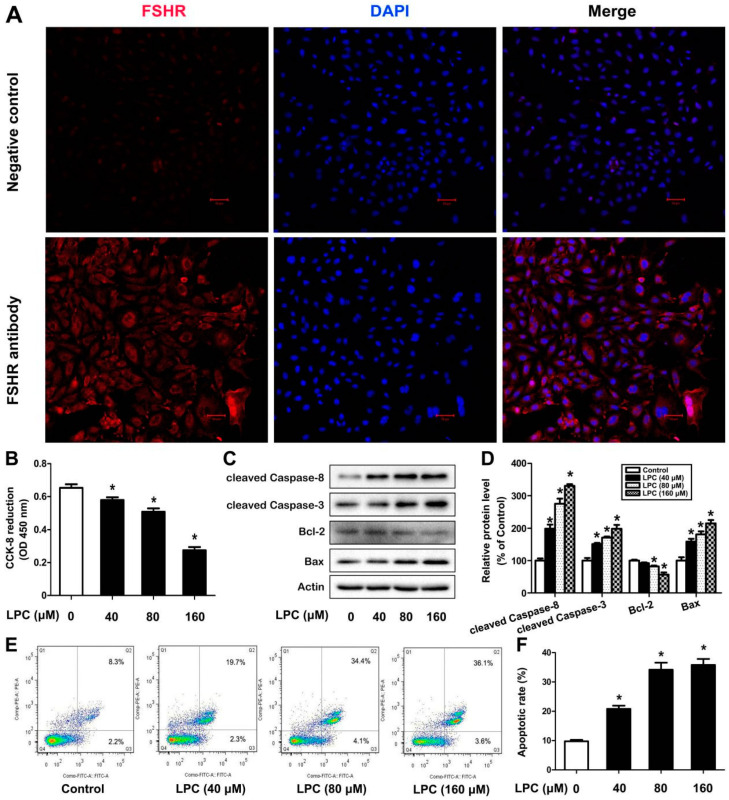
LPC induces apoptosis of mouse ovarian granulose cells. (**A**) Ovarian granulosa cells were isolated from 14-day-old Kunming female mice and the follicle-stimulating hormone receptor (FSHR) was used for identification of cell purity, and the nucleus was stained by DAPI. Negative control was performed without FSHR antibody. The cells were treated with 0–160 μM LPC for 24 h; then cell viability (**B**), the contents of cleavedcaspase-8, cleavedcaspase-3, Bcl-2 and Bax (**C**,**D**), and the annexin V-positive staining cells (**E**,**F**) were detected by the CCK-8 assay, Western blot and flow cytometry, respectively. * *p* < 0.05.

As a specific marker of ovarian granulosa cells, FSHR is utilized to identify the purity of isolated ovarian granulosa cells (GCs). As can be observedin Figure 1A, FSHR was mainly located in the cell membrane of the cells and the purity of GCs was up to 90%, which was enough for subsequent experiments.

To verify whether LPC has a toxic effect on GCs, cell viability was detected after the cells were exposed to 0–160 μM LPC for 24 h. We found that LPC inhibited cell viability in a dose-dependent manner (Figure 1B). Meanwhile, LPC could cause a significant increase in the protein levels of cleaved caspase-3, cleaved caspase-8 and Bax, as well as a decrease in the protein level of Bcl-2 (Figure 1C,D). Furthermore, the amount of early apoptotic cells and late apoptotic cells increased (Figure 1E,F). These results suggested that LPC could induce apoptosis of mouse ovarian granulosa cells.

### 2.2. LPC Induces Autophagy of Mouse Ovarian Granulosa Cells

**Figure 2:**
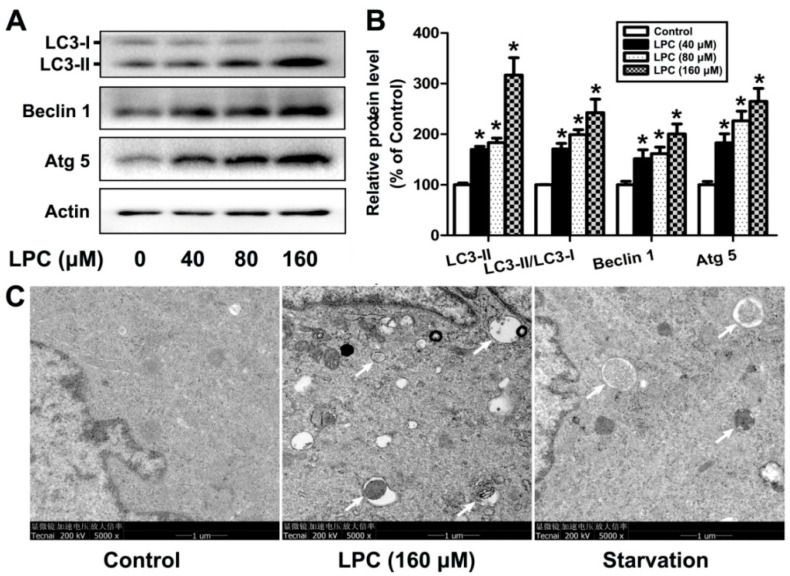
LPC induces autophagy of mouse ovarian granulosa cells. (**A**) The cells were treated with 0–160 μM LPC for 24 h; then the protein levels of Beclin1, Atg5 and LC3 were detected by Western blot. (**B**) The relative protein levels of Beclin1, Atg5 and LC3. (**C**) The cells were treated with 0 or 160 μM LPC for 24 h; then the number of autophagic vacuoles was detected by transmission electron microscopy. Starvation, a positive control group, was induced by treatment with Earle’s balanced salt solution (EBSS) for 2 h. The autophagic vacuoles are pointed out by arrows. * *p* < 0.05.

To investigate whether autophagy is involved in LPC-caused damage on GCs, the levels of autophagy-related proteins, such as Beclin1, Atg5 and LC3, were determined after the cells were exposed to 0–160 μM LPC for 24 h. As shown in Figure 2, LPC significantly increased the protein levels of Beclin1, Atg5 and LC3-II, as well as the ratio of LC3-II/LC3-I. Furthermore, transmission electron microscopy (TEM), a gold standard for detecting autophagy, showed that the number of autophagic vesicles significantly increased in the LPC-treated cells, although quantitative analysis was not conducted due to the failure to obtain enough TEM images. These results indicated that LPC could induce autophagy of mouse ovarian granulosa cells.

### 2.3. LPC Induces Oxidative Stress in Mouse Ovarian Granulosa Cells

**Figure 3:**
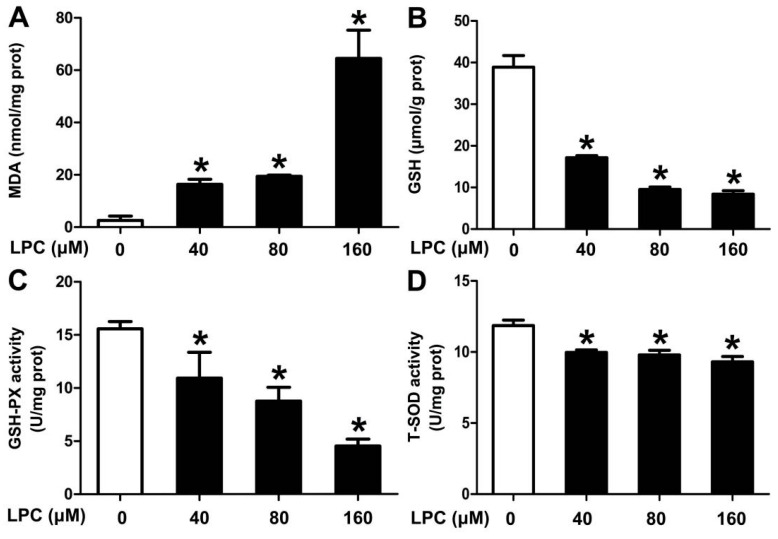
LPC induces oxidative stress of mouse ovarian granulosa cells. The cells were treated with 0–160 μM LPC for 24 h; then the contents of MDA (**A**) and GSH (**B**) and the enzyme activities of GSH-PX (**C**) and SOD (**D**) were detected. * *p* < 0.05.

To figure out the role of oxidative stress in LPC-treated cells, oxidative stress-related indicators, such as MDA, GSH, SOD and GSH-PX, were detected. We found that the content of MDA, indicating membrane lipid peroxidation, enhanced in the LPC-treated cells, while the content of GSH and the activities of GSH-PX and SOD, acting as antioxidant enzymes, decreased (Figure 3). These results indicated that LPC could induce oxidative stress of mouse ovarian granulosa cells.

### 2.4. Oxidative Stress is Involved in LPC-Induced Apoptosis

**Figure 4:**
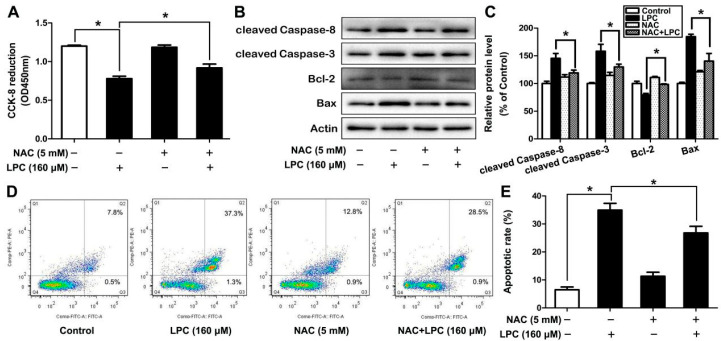
Oxidative stress is involved in LPC-induced apoptosis of mouse ovarian granulosa cells. The cells were treated with 160 μM LPC for 24 h in the presence or absence of 5 mM NAC, then the cell viability (**A**), the protein levels of cleaved caspase-8, cleaved caspase-3, Bax and Bcl-2 (**B**,**C**), and the number of annexin V-FITC-positive cells (**D**,**E**) were observed by CCK-8, Western blot and flow cytometry, respectively. * *p* < 0.05.

An expanding body of studies has shown that oxidative stress is closely related to apoptosis; however, no study shows that oxidative stress plays a role in LPC-induced apoptosis. Herein, GCs were exposed to 160 μM LPC in the presence or absence of 5 mM NAC, an inhibitor of oxidative stress. We found that the inhibition of cell viability and the induction of apoptosis could be alleviated when oxidative stress was inhibited by NAC (Figure 4). These results suggested that LPC induced apoptosis of ovarian granulosa cells via oxidative stress.

### 2.5. Oxidative Stress Is Involved in LPC-Induced Autophagy

**Figure 5:**
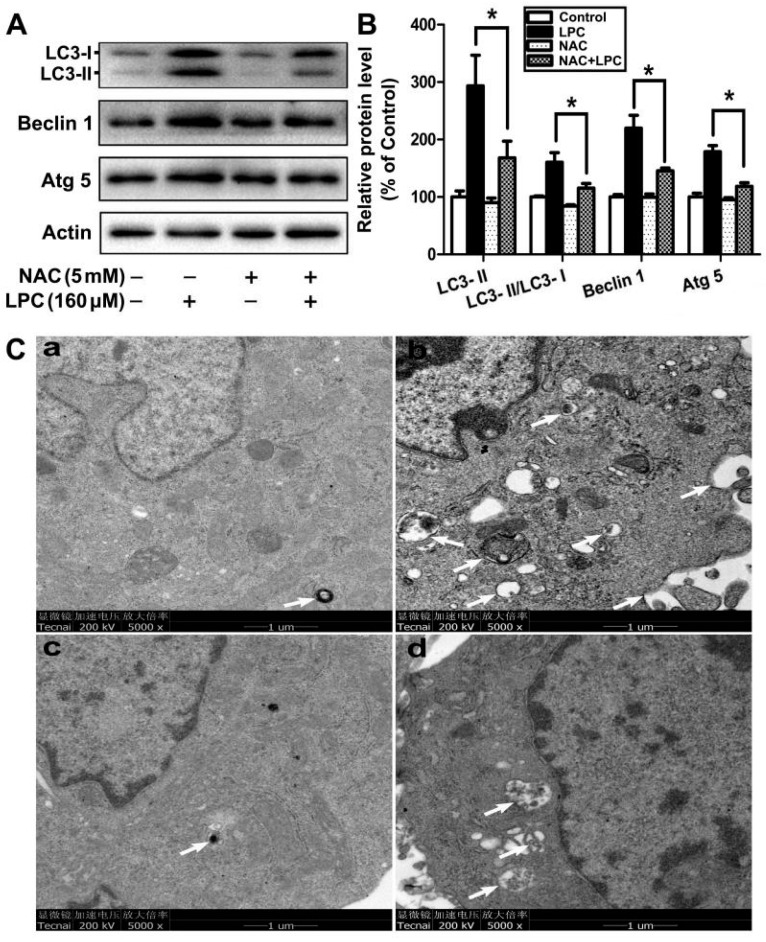
The role of oxidative stress in LPC-induced autophagy of mouse ovarian granulosa cells. (**A**) The cells were treated with 160 μM LPC for 24 h with or without 5 mM NAC; then the protein levels of Beclin1, Atg5 and LC3 were detected by Western blot. (**B**) The relative protein levels of Beclin1, Atg5 and LC3. (**C**) The autophagic vacuoles were observed by transmission electron microscopy after the cells were treated with control (**a**), 160 μM LPC (**b**), 5 mM NAC (**c**) or 5 mM NAC plus 160 μM LPC (**d**) for 24 h. The autophagic vacuoles are pointed out by arrows. * *p* < 0.05.

To find out whether oxidative stress participates in LPC-induced autophagy of mouse ovarian granulosa cells, the cells were treated with 160 μM LPC for 24 h, with or without 5 mM NAC supplement. Compared with the LPC-treated group, the contents of autophagy proteins, such as Beclin1, Atg5 and LC3, were decreased in the LPC plus NAC group (Figure 5A,B). Although accurate quantification result was difficult to obtain because of the lack of enough TEM images, NAC was still shown to decrease the number of autophagic vesicles in the LPC-treated cells (Figure 5C). Taken together, oxidative stress plays a vital role in LPC-induced autophagy of mouse ovarian granulosa cells.

### 2.6. Autophagy Affects LPC-Induced Apoptosis in Mouse Ovarian Granulosa Cells

**Figure 6:**
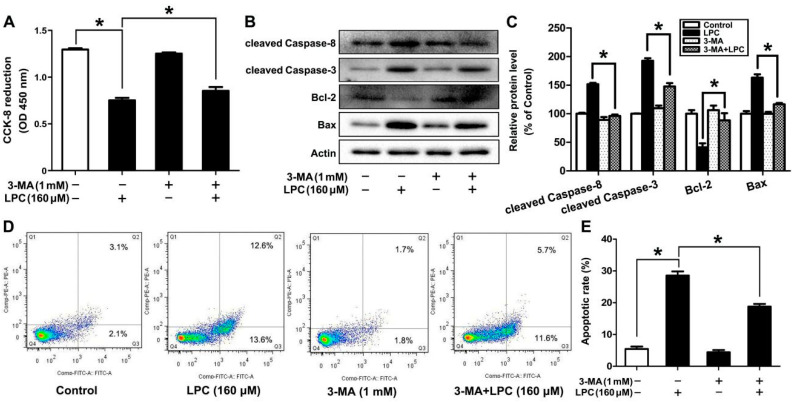
Inhibition of autophagy alleviates LPC-induced apoptosis in mouse ovarian granulosa cells. Mouse ovarian granulosa cells were treated with 160 μM LPC for 24 h with or without 1 mM 3-MA, then the cell viability (**A**) and the protein levels of cleaved caspase-8, cleaved caspase-3, Bax and Bcl-2 (**B**) were detected by CCK-8 assay and Western blot, respectively. (**C**) Densitometry was used to quantify the relative protein levels. (**D**,**E**) Flow cytometry was used to count the number of annexin V-FITC(+)/PI(-) and annexin V-FITC(+)/PI(+) cells. * *p* < 0.05.

In view of the aforementioned information, the interaction between apoptosis and autophagy is discussed.In this study, GCs were treated with 160 μM LPC for 24 h, in the presence or absence of 3-MA (1 mM), which can block autophagy. Compared to the LPC-treated group, the inhibition of cell viability and induction of apoptosis by LPC could be alleviated by 3-MA (Figure 6). In brief, autophagy promoted LPC-induced apoptosis in mouse ovarian granulosa cells.

## 3. Discussion

In recent years, as environmental pollution has become more and more serious, the incidence of female reproductive system-related diseases has increased, so research on the female reproductive system is increasingly valued by scientists. As the main organ of the female reproductive system, the basic unit of the ovary is the follicle, which is surrounded by granulosa cells and plays a key role in the progression of the ovarian cycle [21]. Granulosa cells have been proven to be the initiators of follicular atresia. In the development of follicular atresia, a large number of granulosa cells will undergo apoptosis, then the granular layer is severely destroyed, and the number of follicles is also reduced [22]. Ovarian granulosa cells are closely related to the function of the female reproductive system, and are also the target cells of many toxic substances. In order to obtain mouse ovarian granulosa cells with higher purity, we used mechanical separation to isolate granulosa cells from the ovaries of 14-day-old female Kunming mice; the purity of the granulosa cells was determined by immunofluorescence staining with follicle-stimulating hormone receptor (FSHR) that specifically expresses in the granulosa cells. Compared with the negative control group, the purity of the isolated primary granulosa cells can meet the needs of the experiment.

As an endogenous inflammatory phospholipid, LPC can be produced under both physiological and pathological conditions, and exerts different effects on the body. It is beneficial to human health under physiological conditions and participates in many diseases under pathological conditions, such as bronchial asthma and atherosclerosis. In addition, LPC, as one of the ingredients of cosmetics and ointments, is toxic to the skin [23]. After treating human umbilical vein endothelial cells (HUVECs) with a series of concentrations of LPC for 24 h, LPC causes damage to HUVECs, which proves that LPC is cytotoxic [24]. Our study has found that LPC decreased the viability of granulosa cell in a concentration-dependent manner, and when the concentration of LPC was 160 μM, the cell viability was decreased by 50%, suggesting that LPC has a toxic effect on mouse ovarian granulosa cells.

As one of the terminal pathways of cell death, apoptosis is a kind of self-regulated, programmed cell death, which can balance the survival and death of cells and maintain normal tissue homeostasis [25]. LPC can make the cells have typical apoptotic morphology changes, such as cytoplasmic vacuoles, mitochondrial swelling, and the biochemical characteristics of apoptosis, including loss of mitochondrial membrane potential, release of cytochrome C, up-regulation of caspase-3, PARP-1, Bax protein level and decrease in Bcl-2 level [26]. The initiator and performer of the caspase cascade are caspase-8 and caspase-3, respectively. The cleaved caspase-8 and caspase-3 will increase when apoptosis occurs [27]. Bcl-2 family members play an important role in mitochondrial-dependent caspase cascade activation. As the core members of the Bcl-2 family, Bax and Bcl-2 play an important role in regulating apoptosis [28,29]. In this study, mouse ovarian granulosa cells were treated with 0, 40, 80, and 160 μM LPC for 24 h, and then Western blot was used to detect the changes in apoptosis-related protein levels in the cells. We found that the expression of cleaved caspase-8, cleaved caspase-3 and Bax significantly increased, while the expression of Bcl-2 significantly reduced in the LPC-treated cells, suggesting that LPC induced apoptosis of mouse ovarian granulosa cells.The results of the annexin V-FITC/PI double staining detection method showed that the rate of apoptosisof the cells increased with increasing LPC concentrations, which further verified that LPC can induce apoptosis of mouse ovarian granulosa cells.

Autophagy can maintain the energy balance and function of cells, and control the fate of cells through different crosstalk signals. At the same time, it is also considered to be a kind of programmed cell death, namely, type II programmed cell death [30]. As a key molecular regulator of autophagy, Beclin1 interacts with several cofactors, such as Atg14L, PINK and survivin, to promote the formation of the complex Beclin1–Vps34–Vps15, thereby inducing autophagy [31]. Autophagy is mainly performed by the autophagy-related (ATG) gene family, and ATG-5 is one of the most important regulators in the induction of autophagy [32]. The production of LC3-II depends on Atg5 and its combination with Atg12. The C-terminal fragment of LC3-II can be cleaved to produce LC3-I, called the cytoplasmic form, and the transformation characteristics of LC3-II to LC3-I can be used to monitor the activity of autophagy, and the ratio of LC3-II or LC3-II/LC3-I is related to the number of autophagosomes [33]. In our study, the expression of autophagy-related proteins, such as Atg5, Beclin1, and LC3-II, and the ratio of LC3-II/LC3-I increased significantly after the cells were treated with LPC for 24 h, suggesting that LPC can induce autophagy in mouse ovarian granulosa cells, which is further verified by the increased number of autophagic vesicles found in the cells using TEM examination.

Oxidative stress refers to a state of imbalance between the production of reactive oxygen species and antioxidant defense in the body. Its essence is that the dynamic balance between biological antioxidants and reactive oxides is broken, which, in turn, causes the level of ROS to increase [34]. Common antioxidant enzymes include catalase, superoxide dismutase (SOD), glutathione peroxidase (GSH-PX) and peroxidase III (PrxIII). Malondialdehyde (MDA) is an indicator that indirectly reflects the degree of cellular oxidative damage, and is widely used in the assessment of oxidative stress [35]. LPC has been proven to be an effective ROS-promoting factor, which can activate the ERK1/2 signal by generating mitochondrial ROS in endothelial cells [36]. In human umbilical vein endothelial cells, LPC can induce oxidative stress, accompanied by a decrease in the content of SOD and the enzyme activity of GSH-PX, and an increase in the content of MDA and ROS [26]. We found that the MDA content in the mouse ovarian granulosa cells increased significantly, and the GSH content and the activities of SOD and GSH-PX significantly reduced after the cells were treated with 0–160 μM LPC for 24 h, which verified that LPC can induce oxidative stress on GCs.

Abnormally elevated ROS can damage proteins, lipids and DNA in cells, lead to disruption of genes, cell structure and function, and, ultimately, the induction of cell apoptosis. Chang et al. showed that LPC can induce apoptosis by inducing an increase in the level of ROS in endothelial cells [37]. Oxidative stress is also one of the reasons for the induction of apoptosis in mouse ovarian granulosa cells [38]. The latest research shows that LPC can induce microglia to produce ROS and induce autophagy in a demyelinated mouse model [39]. In the current study, NAC, an inhibitor of oxidative stress, was shown to alleviate the induction of apoptosis and autophagy of mouse ovarian granulosa cells by LPC, which verified that LPC-induced oxidative stress mediates apoptosis and autophagy of mouse ovarian granulosa cells.

More and more evidence shows that there is cross-talk between autophagy and apoptosis, although the related signal transduction pathways are different. Autophagy has a cytoprotective effect under certain stress conditions, and can reduce cell death by inhibiting apoptosis. In other cases, autophagy is another death pathway that can lead to increased cell death [40]. Yang et al. shows that ZnO NP-induced autophagy can promote the induction of apoptosis in mouse GC-1 spg cells [41]. Autophagy can also promote the induction of apoptosis in mouse spermatogonia cells by DEHP [42]. In order to investigate the contribution of autophagy in the LPC-induced apoptosis of mouse ovarian granulosa cells, the cells were exposed to LPC for 24 h by pretreatment with or without 1 mM 3-MA for 30 min. We found that LPC-induced apoptosis of ovarian granulosa cells was significantly alleviated when autophagy was inhibited, which proved that LPC-induced autophagy can promote the induction of apoptosis in mouse ovarian granulosa cells.

## 4. Materialsand Methods

### 4.1. Materials and Reagents

LPC (830071P), N-acetyl-L-cysteine (NAC) and 3-methyladenine (3-MA) were purchased from Sigma (St. Louis, Missouri). Rabbit anti-follicle-stimulating hormone receptor (FSHR) polyclonal antibody (ab150557) was purchased from Abcam (Cambridge, MA, USA). CCK-8 was purchased from Dojindo Laboratories (Tokyo, Japan). The apoptosis-related antibodies, including rabbit anti-caspase-8 polyclonal antibody (sc-7890), rabbit anti-caspase-3 polyclonal antibody (sc-7148), mouse anti-Bcl-2 monoclonal antibody (sc-492) and rabbit anti-Bax antibody (sc-493), were bought from Santa Cruz Biotechnology (Santa Cruz, CA, USA). Rabbit anti-LC3 polyclonal antibody (PD014), rabbit anti-ATG5 polyclonal antibody (PM050) and rabbit anti-Beclin 1 polyclonal antibody (PD017) were obtained from MBL Co. Ltd. (Nagoya, Japan). Malondialdehyde (MDA) assay kit (A003-1-2), superoxide dismutase (SOD) assay kit (A001-3-2), glutathione (GSH) assay kit (A006-2-1) and glutathione peroxidase (GSH-PX) assay kit (A005-1-2) were bought from Nanjing Jiancheng Bioengineering Institute (Nanjing, China). The Annexin V-FITC Apoptosis Kit was obtained from Invitrogen Life Technologies (Carlsbad, CA, USA).

### 4.2. Isolation of Mouse Ovarian Granulosa Cells

According to the procedure described in our previous study [43], ovaries were isolated from 14-day-old female mice, and the extra tissue and capsule around the ovaries were removed. Then the ovaries were treated with trypsin, IV collagenaseand pierced by a needle in order to release mouse ovarian granulosa cells. After incubation at 37 °C for 15 min, DMEM/F-12 medium supplemented with 10% fetal bovine serum (FBS) was added to inhibit the activity of enzymes. Supernatant was discarded after the medium containing the cells was centrifugated at 1500 rpm for 5 min, and the cells in the lower layer of centrifuge tube were cultured in DMEM/F-12 medium supplemented with 10% FBS, 100 IU/mL penicillin and 100 μg/mL streptomycin under 37 °C, 5% CO_2_ condition.

### 4.3. The FSHR Fluorescence Staining

Isolated mouse ovarian granulosa cells were planted on cell slides; the cells were then fixed with 4% paraformaldehyde and blocked in 3% BSA for 1 h at room temperature. After being washed by PBS three times, the cells were incubated with rabbit anti-FSHR polyclonal antibody (1:100 dilution) overnight at 4 °C. The cells were then incubated with rhodamine/TRITC-conjugated secondary antibodies (1:200 dilution) for 15 min after being washed by PBS three times. Nucleus was stained with DAPI (Invitrogen, Waltham, MA, USA). The pictures were presented with a fluorescence microscope (Nikon TE-2000, Tokyo, Japan).

### 4.4. Cell Counting Kti-8(CCK-8) Assay

Mouse ovarian granulosa cells were seeded in a 96-well plate; the cells were then treated with the indicated concentrations of LPC (0, 40, 80 and 160 μM) with or without NAC (5 mM) or 3-MA (1 mM) for 24 h. Following this, 10 μL CCK-8 was added to each well, the absorbance of each well was then measured with a spectrophotometer at 450 nm after incubation at 37 °C for 2 h.

### 4.5. Western Blotting Analysis

After pretreatment with or without NAC (5 mM) or 3-MA (1 mM), the cells were treated with LPC at the indicated concentration of 0–160 μM for 24 h. Then, the cells were collected and harvested in RIPA lysis buffer with cocktail protease inhibitor. After 4×protein loading buffer was mixed with samples, each group containing the same loading amount was detected by Western blot analysis.

### 4.6. Oxidative Stress Measurement

Mouse ovarian granulosa cells were treated with 0–160 μM LPC for 24 h. After they homogenized, the cells were centrifuged at 800× *g* for 10 min and the supernatants were used to determine the contents of MDA and GSH, as well as the enzyme activities of SOD and GSH-PX. The above operations were carried out according to the manufacturer’s instructions.

### 4.7. AnnexinV-FITC/PI Apoptosis Assay

After treatment with 0–160 μM LPC in the presence or absence of NAC (5 mM) or 3-MA (1 mM) for 24 h, the cells were collected after being centrifuged at 500× *g* for 5 min. The cells were resuspended in 1 × buffer and incubated with annexin V-FITC and propidium iodide (PI) for 15 min in a dark environment before testing.

### 4.8. Transmission Electron Microscopy Analysis

The collected cells were fixed in ice-cold 2.5% glutaraldehyde after being centrifuged (1000 rpm for 10 min). Next, fixed cells were treated according to the instructions in our previous study [44]. The samples were observed by transmission electron microscope (Hitachi H800).Representative TEM image is presented in the Results section due to failure inobtaining an accurate quantification result.

### 4.9. Statistical Analysis

All results were represented as means±standard deviation. The statistical differences between groups were evaluated by one-way ANOVA. Each experiment was repeated in triplicate. *p* < 0.05 presented significant differences among groups.

## 5. Conclusions

Taken together, LPC had a toxic effect on mouse ovarian granulosa cells, and LPC-induced apoptosis and autophagy was due to the induction of oxidative stress. Furthermore, autophagy could promote LPC-induced apoptosis of mouse ovarian granulosa cells.

## Data Availability

Not applicable.
